# Mediational Model of Multiple Sclerosis Impairments, Family Needs, and Caregiver Mental Health in Guadalajara, Mexico

**DOI:** 10.1155/2018/8929735

**Published:** 2018-01-17

**Authors:** Melody N. Mickens, Paul B. Perrin, Adriana Aguayo, Brenda Rabago, Miguel A. Macías-Islas, Juan Carlos Arango-Lasprilla

**Affiliations:** ^1^Department of Psychology, Virginia Commonwealth University, Richmond, VA, USA; ^2^Department of Physical Medicine & Rehabilitation, Virginia Commonwealth University School of Medicine, Richmond, VA, USA; ^3^Department of Neuroscience, CUCS, University of Guadalajara, Guadalajara, JAL, Mexico; ^4^Department of Psychology, Enrique Diaz de Leon University, Guadalajara, JAL, Mexico; ^5^BioCruces Health Research Institute, Cruces University Hospital Barakaldo, Bizkaia, Spain; ^6^IKERBASQUE, Basque Foundation for Science, Bilbao, Spain

## Abstract

Individuals with multiple sclerosis (MS), especially those living in Latin America, often require assistance from family caregivers throughout the duration of the disease. Previous research suggests that family caregivers may experience positive and negative outcomes from providing care to individuals with MS, but few studies have examined the unmet needs of individuals providing care to family members with MS and how these unmet needs may mediate the relationship between MS symptoms and caregiver mental health. The current study examined the relationships among MS impairments (functional, neurological, cognitive, behavioral, and emotional), unmet family needs (household, informational, financial, social support, and health), and caregiver mental health (satisfaction with life, anxiety, burden, and depression) in a sample of 81 MS caregivers from Guadalajara, Mexico. A structural equation model demonstrated the mediational effect of unmet family needs on the relationship between MS impairments and caregiver mental health. These findings suggest that intervention research on MS caregivers in Latin America may consider focusing on caregiver mental health problems by addressing unmet family needs and teaching caregivers ways to manage the impairments of the individual with MS.

## 1. Introduction

Multiple sclerosis (MS) is a chronic neurological illness that eventually results in physical disability and cognitive impairments which limit an individual's ability to function independently [1]. Approximately 2.5 million people have been diagnosed with MS worldwide [2], and research demonstrates that worldwide prevalence rates are increasing [3]. As with other countries, researchers have observed that MS prevalence rates may be higher than previously reported in Latin American countries such as Mexico, where current prevalence rates vary by region and range from 7 to 30 cases per 100,000 people [4, 5].

In Latin America, where rates of MS are increasing but disparities still exist in its diagnosis and treatment [6, 7], sociocultural values such as allocentrism, familism, and filial obligation [8, 9] increase the likelihood that family members will serve as informal caregivers to individuals with MS. When compared to other racial/ethnic groups, Latino caregivers often report limited use of formal support services [10, 11], larger informal social networks [8], increased role strain [12], lower rates of institutionalization [10], and higher rates of depression [13]. However, very few studies have examined MS caregiving in Latin America [14, 15], and associations in this region among MS impairments, needs of family members providing care, and caregiver mental health remain largely unknown. Because of this major gap in the research literature, there is a great need for research addressing the process of MS caregiving in Latin America, as well as the impact of MS impairments on family needs and caregiver mental health in this region.

International research has established that compared to noncaregivers, MS caregivers report higher levels of depression [16], anxiety [17], and decreased social support [18]. Patient factors such as level of disability [19–21], cognitive impairments [22–25], behavioral changes [22, 23], incontinence, and fatigue [26] contribute to increased caregiver depression, strain, and burden. Although many of the findings on MS caregiver functioning emphasize the negative aspects of caregiving, the literature also demonstrates that MS caregivers report salubrious outcomes such as personal growth, role fulfillment, positive emotions, and satisfaction as a result of caregiving [11, 27].

MS caregiving can be understood using Pearlin et al.'s [28] conceptual model of caregiver stress. This model identifies three domains of caregiving stress: (a) background and context of the caregiving situation (i.e., caregiver age, gender, ethnicity, socioeconomic status, relationship with the patient, and family and social network composition), (b) primary stressors (i.e., cognitive functioning of patient, behavioral changes, problematic behaviors of the patient, activities of daily living (ADLs), and instrumental ADLs), and (c) secondary stressors (i.e., unmet needs for information, reduced access to employment or need for financial assistance, limited social support, family conflict, conflict with occupational and social role fulfillment, economic strain, changes in self-concept, loss of self, role captivity, mastery, competence, and gain). The model identifies physical and emotional outcomes associated with stressors and alleviated by mediators of stress (i.e., coping strategies and social support). Moreover, Pearlin et al. indicate that stressors, mediators, and outcomes often interact and individually or collectively influence caregiver mental health in a direct, indirect, or cyclical pattern [28]. Because unmet family needs can comprise both background (i.e., affecting family prior to involvement in caregiving roles) variables and stressors associated with the caregiving experience (i.e., lack of knowledge about disease process or need for specific care information), clinicians and providers have increased interest in studying the role of unmet needs on caregiver psychological functioning.

Findings from studies of MS caregivers demonstrate that primary stressors (i.e., patient functioning) have been associated with depression and burden. Although these studies have examined aspects such as the patient's cognitive functioning, psychological functioning, physical disability, and ADL impairments, few have examined unmet family needs in the context of these patient-related stressors. Within the framework of Pearlin et al.'s model [28], family needs (i.e., household needs, informational needs, financial needs, health needs, and social support) are an extension of the background/context and secondary stressor domains. Findings from a previous study demonstrate that unmet family needs are also are a central determinant of caregiver adjustment, as they have been associated with increased burden and depression among MS caregivers [14].

Given the often significant impairments documented in individuals with MS, the unknown levels of unmet family needs, and the generally poor mental health that MS caregivers report, many questions remain regarding the specific connections among these sets of variables, especially in Latin America. As such, the objective of the present study is to examine unmet family needs as a mediator of the established relationship between the care recipients' MS impairments (primary stressor) and caregivers' mental health (outcome). Based on prior research which suggests strong associations between patients' MS symptoms and their caregivers' psychosocial distress [16, 17, 19, 22, 24], it is hypothesized that the relationship between MS impairments and caregiver mental health will be significantly mediated by unmet family needs. At present, no studies in the MS caregiver literature have examined this possible effect, but based on Pearlin et al.'s [28] model, primary stressors (e.g., patient functioning) should be associated with reduced caregiver mental health outcomes. Secondary stressors such as family needs should be associated with both primary stressors (MS impairments) and negative caregiver mental health outcomes and could possibly account for the connection between these two sets of constructs.

## 2. Method

### 2.1. Participants

Participants (*n* = 81) were a convenience sample of self-identified MS caregivers recruited from The Mexican Foundation for Multiple Sclerosis and the Department of Neurosciences of the University Center for Health Sciences, University of Guadalajara, Mexico. In order to participate in the study, caregivers had to (a) be the primary caregiver of an individual with a diagnosis of MS who was at least six months past the date of diagnosis, (b) have provided care to the person with MS for a minimum of six months, and (c) have had no history of a cognitive, serious psychiatric, or neurological disorder themselves. Initially, 86 participants were approached, but after screening, five declined or did not meet study criteria. Data were collected from a final sample of 81 caregivers. Demographic information for the caregiver sample is provided in Table 1 and for the patient sample in Table 2.

### 2.2. Measures

Eligible caregivers completed a battery of questionnaires in Spanish that assessed the following domains: demographic information, MS-related impairments as observed by the caregiver, family needs, and mental health. Measures of depression, anxiety, caregiver burden, satisfaction with life, and caregiver needs had been previously translated to Spanish and validated in Spanish-speaking samples prior to their use in this study. Spanish-speaking norms were used for scoring and interpreting these measures where available. The measure of MS impairments was translated (forward and backward) into Spanish and then English using methods published by Chapman and Carter [29] and Guillemin et al. [30] to ensure cross-cultural equivalence. Both translations were compared by a monolingual psychologist from Mexico and a bilingual psychologist living in Spain. No discrepancies were identified. The final version was reviewed by the monolingual psychologist from Mexico. Participants completed a demographic form created by the investigators. On this form, household income in Mexico was calculated by monthly salary, where the monthly minimum wage at the time of data collection was 2018.70 pesos or approximately 155.40 USD per month [31].

#### 2.2.1. MS Impairments

Caregivers completed the MS Impairment Questionnaire (MS-IQ) [26], a 30-item checklist of common MS impairments. Assessed impairments are grouped into five subscales: cognitive, emotional, behavioral, neurological, and functional. Caregivers completed this measure by reporting “yes” for the specific impairments that their care recipient experienced and “no” for the impairments that the care recipient did not experience. Item scores are summed (yes = 1, no = 0) so that subscale scores with higher values indicate domains with a larger number of impairments. Although it is possible to calculate a total score, only the subscale scores were used in the current study in order to generate a latent construct.

#### 2.2.2. Anxiety

Caregivers completed the Spielberger State-Trait Anxiety Inventory (STAI) [32] as a measure of anxiety. The STAI is a 40-item self-report measure with a two-factor structure. The S-anxiety subscale measures anxiety as a temporary emotional state, while the T-anxiety subscale assesses anxiety as a fixed personality trait [32, 33]. Both subscales can be combined to create a total scale which was used in the current study. Total scale scores range from 40 to 160, with higher scores indicating increased anxiety. The Spanish version of the STAI [34] was used in this study and has demonstrated very good construct validity and internal consistency in samples of male (state *α* = 0.93, trait *α* = 0.96) and female (state *α* = 0.88, trait *α* = 0.82) Spanish speakers [35, 36].

#### 2.2.3. Burden

Caregivers completed the Zarit Burden Inventory (ZBI) [37]. Item scores are summed, and total scores range from 0 to 88 with higher scores indicating greater burden [38]. The ZBI has been validated and used in numerous neurological caregiver populations including TBI caregivers [39], dementia caregivers [37], and Parkinson's caregivers [40]. The Spanish version of the ZBI has demonstrated excellent construct validity and internal reliability (*α* = 0.92) in samples of Spanish-speaking individuals [41].

#### 2.2.4. Depression

Caregivers completed the Patient Health Questionnaire-9 (PHQ-9) [42] as a measure of depressive symptoms experienced within a two-week period. Total scores range from 0 to 27 with higher scores reflecting more severe symptoms of depression. The Spanish version has demonstrated strong construct and criterion validity, as well as excellent internal consistency and convergent validity in Spanish-speaking validation samples [43–45].

#### 2.2.5. Satisfaction with Life

Participants completed the Satisfaction with Life Scale (SWLS) [46]. Higher total scores represent higher life satisfaction [47]. Participants completed the Spanish version of the SWLS, which has high internal consistency (*α* = 0.88) and good construct validity in Spanish-speaking samples [48, 49].

#### 2.2.6. Family Needs

The Family Needs Assessment Tool (FNAT) [50] assesses the degree to which needs are met in family caregivers of individuals with neurological conditions in Latin America. The FNAT is comprised of 14 items and has five unmet needs subscales: household (two items), informational (three items), financial (three items), health (four items), and social support (two items). Higher scores indicate greater areas of unmet needs. As with the MS-IQ, although it is possible to calculate a total score on the FNAT, only the subscale scores were used in the current study in order to generate a latent construct.

### 2.3. Procedure

Prior to recruitment, the Institutional Review Board of the Mexican Foundation of Multiple Sclerosis reviewed and approved the study protocol. Staff at the Mexican Foundation for Multiple Sclerosis and the Department of Neurosciences of the University Center for Health Sciences, University of Guadalajara recruited prospective study participants from a neurology clinic using verbal and written advertisements. Interested participants contacted the research staff and were screened for eligibility. Eligible caregivers completed informed consent forms prior to data collection. During a 40-minute appointment at the Mexican Foundation for Multiple Sclerosis, a staff psychologist collected sociodemographic information and administered a battery of questionnaires to caregivers using a structured interview format to ensure that the participants understood the item content and did not skip any items.

### 2.4. Data Analysis

#### 2.4.1. Preliminary Analyses

Frequencies and descriptive statistics were run to summarize MS impairments reported by caregivers, frequently reported unmet family needs, and clinically significant caregiver mental health problems.

#### 2.4.2. Hypothesis Testing

A structural equation model (SEM) was created with three latent variables: MS impairments, family needs, and caregiver mental health. MS impairments was comprised of shared variance from the five impairment variables: functional, cognitive, behavioral, emotional, and physical. Family needs were comprised of shared variance from the five types of family needs: household, informational, financial, health, and social needs. Caregiver mental health was comprised of shared variance from the four mental health variables: depression, burden, anxiety, and satisfaction with life. This SEM was conducted using AMOS 20 [51]. Because most traditional SEMs in rehabilitation research are run with at least 200 participants [52], and the sample size in the current study is 81 participants, estimates of model fit are likely to be inaccurate; we report indices of model fit solely for reference. Instead, the focus of this analysis was on the size and significance level of the standardized *β* weight for the indirect effect of MS impairments on caregiver mental health through family needs.

## 3. Results

Participants reported patient impairments in all five domains, as seen in Table 3. Of the neurological impairments reported, more than 75% of participants reported tiring easily, while over half reported paralysis, poor eyesight, loss of sensation, and clumsiness. More than half of the sample reported the following emotional symptoms: depression, easily upset, irritability, and mood changes. Commonly reported functional and cognitive impairments were difficulty walking, doing things slowly, forgetfulness, and difficulty concentrating. Less than half of participants reported behavioral symptoms but endorsed acting impulsively as the most commonly observed behavioral symptom. Caregivers' item responses to the FNAT were ranked (identifying the top five) by the percentage of unmet need endorsements. As illustrated in Table 4, a majority of the unmet needs identified were from the informational domain, while the remaining needs were from the social support domain.

Total scores on the PHQ-9 ranged from 0 to 21 out of a possible maximum score of 27. The sample mean of 5.92 (SD = 5.27) indicated frequent endorsement of mild symptoms of depression. As seen in Table 5, nearly half of the sample reported clinically significant levels of depression, with 26% reporting mild symptoms, 16% reporting moderate symptoms, and 1.2% reporting severe symptoms of depression. Both total and subscale scores (e.g., state and trait) of the STAI were examined. Participants' total scores ranged from 11 to 93 out of a maximum score of 160. Nearly one-third of participants reported clinically significant symptoms of state or trait anxiety with 32% reporting moderate symptoms on the state subscale and 2.5% reporting severe symptoms on the state subscale of the measure. Responses on the trait subscale demonstrated that 31% of participants reported moderate symptoms on the trait subscale, while 3.7% reported severe symptoms. Total scores on the ZBI ranged from 0 to 62 out of a maximum score of 88. The sample mean of 22.02 (SD = 14.72) indicated that on average participants reported mild to moderate symptoms of burden. Further review of clinically significant scores revealed that 29.6% reported mild to moderate symptoms, 12.3% reported moderate to severe symptoms, and 1.2% reported severe symptoms of burden. Total scores on the SWLS ranged from 10 to 35 out of a maximum score of 35. The sample mean of 23.43 (SD = 6.35) indicated an overall feeling of general satisfaction.

**Figure 1:**
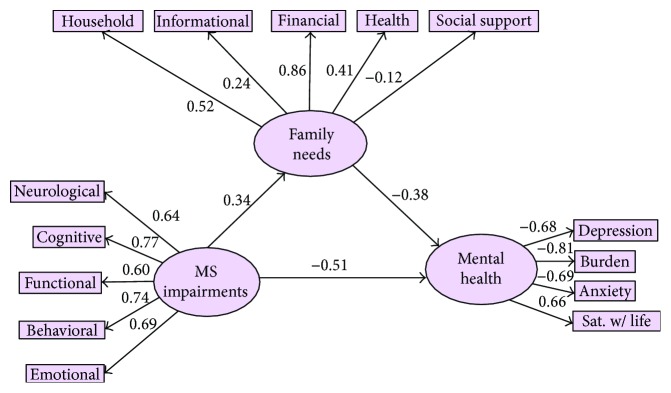
SEM of the mediation of family needs on the relationship between MS impairments and caregiver mental health.

Two structural equation models (SEMs) were created to examine whether unmet family needs mediated the relationship between MS impairments and caregiver mental health. Both models included three latent variables: MS impairments, family needs, and caregiver mental health. MS impairments was comprised of the following five manifest variables (i.e., subscale scores from the MS-IQ): neurological, cognitive, functional, behavioral, and emotional symptoms. Family needs was created using the five manifest variables (i.e., subscale scores from the FNAT) of financial, informational, household, health, and social support needs. Caregiver mental health was created using four manifest variables of depression (PHQ-9 total score), anxiety (total STAI score), burden (total ZBI score), and satisfaction with life (total SWLS score). In total, the models were comprised of 33 variables, of which 14 were observed, 16 were unique, and 3 were factors. The manifest variables are directly measured by a total score or subscale score, represented in Figure 1 (the second, structural model) by rectangles. The latent variables are measured indirectly and inferred mathematically from the shared variance of the manifest variables. In Figure 1, the latent variables are represented by ovals.

Normality tests revealed that the distributions of the measured variables were all normal in a univariate sense in terms of skewness (all coefficients ≤ an absolute value of 0.93) and kurtosis (all coefficients ≤ an absolute value of 1.17). Similarly, a Mardia's coefficient of 2.23 suggested that the variables were not multivariate kurtotic. It was further found by the calculation of Mahalanobis distance that no single observation was meaningfully far from the multivariate centroid (all *p*s ≥ 0.01), and therefore there were no multivariate outliers.

The first measurement model SEM examined correlations (e.g., bidirectional paths) between each of the latent variables as opposed to directional paths. In this model, only one statistically significant correlation emerged between MS impairments and caregiver mental health at *r* = −0.64 (*p* < 0.01). The bivariate relationships between MS impairments and family needs (*r* = 0.34, *p* = 0.39) and family needs and caregiver mental health (*r* = −0.55, *p* = 0.37) were not statistically significant. Although two of these correlations were not statistically significant, all three were in the expected direction and were at least medium sized.

In the second SEM (Figure 1), MS impairments were specified to lead directly to caregiver mental health, as well as to have an indirect effect on caregiver mental health through family needs. In this model, MS impairments was significantly associated with caregiver mental health (*β* = −0.51, *p* = 0.003). MS impairments were not significantly associated with family needs (*β* = 0.34, *p* = 0.39), nor was family needs associated with caregiver mental health (*β* = −0.38, *p* = 0.39). However, again all three directional paths were in the hypothesized direction. The indirect effect of MS impairments on caregiver mental health through family needs was statistically significant (*β* = 0.13, *p* = 0.008), suggesting the presence of an indirect effect. The following fit indices are presented only for reference: the ratio of the *χ*^2^ statistic to the degrees of freedom in the model was 1.59; the goodness of fit index (GFI) was 0.85; the adjusted goodness of fit index (AGFI) and the normed fit index (NFI) were 0.78 and 0.72, respectively; the incremental fit index (IFI), Tucker-Lewis index (TLI), and comparative fit index (CFI) were 0.87, 0.84, and 0.87, respectively; and the root mean square error of approximation (RMSEA) was 0.09.

## 4. Discussion

The present study examined the caregiving experiences of MS caregivers living in Guadalajara, Mexico, with a specific emphasis on identifying unmet family needs as a possible mediator of the relationship between MS impairments and caregiver mental health. As hypothesized, this study's findings supported that unmet family needs mediated the relationship between MS impairments and caregiver mental health.

Prior to this study, several researchers had identified a very strong relationship between patients' clinical symptoms and their caregivers' psychosocial functioning. However, few, if any, researchers have been able to identify specific mechanisms or correlates that account for this relationship. The statistically significant indirect effect of MS impairments on caregiver mental health through unmet family needs is the first time this finding has emerged in the research literature. One possible interpretation of this finding is that as patients experience impairments in multiple domains, family caregivers may need additional support or may have new needs that they did not have when the patient's health was more stable [53]. When these needs are unmet, the family has fewer coping resources to draw upon and family members may experience greater distress. Previous research on caregivers of individuals with moderate to severe MS impairments has consistently identified increased needs for social, informational, and financial support, as well as higher rates of burden, strain, and depression [22, 54–57].

Findings from this study suggest that parents are providing the majority of the care for their children. This expands the definition of MS caregiver often seen in previous studies from a singular perspective that includes a spouse or romantic partner to a broader definition that can include parents, siblings, or other individuals providing care to the patient within their family system. As such, health care providers may need to focus on the impact of the patient and their illness on family overall. Consideration for the needs of the family system is particularly warranted in this sample as the overall health of the family unit is an important part of Mexican and Latino American cultures. Because unmet family needs were identified as a mediator of the relationship between MS impairments and caregiver mental health, health care providers may also want to assess the psychosocial functioning of family members in the household and target interventions toward the family system. There is the potential for a multifamily group intervention that informs caregivers about the effects of caregiving on families. Such an intervention may help bring together families within the community and could help normalize feelings of burden, disappointment, guilt, and fear that caregivers may be too guarded to share with others.

With regard to primary unmet needs identified in this study, a large number of participants reported unmet informational needs, which included specific requests for “specialized information about the patient,” “complete information,” and “to share [their] feelings with someone who has been in the same situation.” Caregivers in this sample may benefit from general education about MS (e.g., disease course, symptom types, and treatments), as well as specific information about behavioral and emotional impairments (e.g., psychoeducation, resource identification, and symptom management strategies). Data from caregivers of individuals with Alzheimer's disease [58] and Parkinson's disease [59] have demonstrated decreased burden and caregiver stress among individuals who complete psychoeducational programs for management of symptoms associated with these illnesses [60]. Likewise, because of the specific interest identified by these caregivers, MS caregivers could possibly benefit from education that assists with daily care needs or helps caregivers understand and anticipate possible sequelae of the disease.

Additionally, the caregivers may have had informational needs that were not assessed. As such, a focus group or follow-up survey to assess the type of information that caregivers need is a feasible way to help meet the needs of caregivers in this sample or other samples as different caregiver cohorts will likely have different needs dependent on the patient's course, family composition, and family's access to medical resources. Moreover, although caregivers were requesting information in this study, they indicated a need to speak with other caregivers and to receive social support in addition to pragmatic suggestions, recommendations, and information. Interventions designed to facilitation dissemination of information through peer support, mentoring, and support groups offered in person or via telephone or internet have demonstrated efficacy in other caregiver populations [61] and could provide MS caregivers with increased access to these resources as well.

Interventions designed to help caregivers determine how to meet financial and household needs could help alleviate the negative mental health outcomes that caregivers in this sample experienced. Addressing these needs may include education about resources in the community (e.g., grants, supplemental income, and respite care services) that are available to families in the region or education about how to delegate household tasks. Addressing these needs may also require education about when to seek support outside of the family (e.g., respite care) and ways to overcome cultural barriers to accepting and accessing care outside of a kinship network.

In addition to this key finding, mental health outcomes reported by this sample differed somewhat from other studies of MS caregivers and individuals in Mexico. Epidemiological data from Mexico suggest a lifetime prevalence of 7.2% for major depressive disorder [62], which demonstrates that reported rates of depression in the current study are higher than those generally reported in the Mexican population. Although few studies have examined anxiety among MS caregivers, Argyriou et al. [63] reported mean scores that reflected mild anxiety in their sample, while the scores in the current study demonstrated subclinical mean values for state and trait anxiety, even though one-third of the sample endorsed moderate symptoms of anxiety. When comparing this sample's scores to epidemiological lifetime prevalence data for anxiety in Mexico, participants in this sample reported higher than expected anxiety (i.e., 14.3% prevalence rate as reported by Medina-Mora et al. [62]). A comparison with other studies of MS caregivers demonstrates that the current sample's percentages of MS caregivers experiencing mild, moderate, and severe burden are much lower than rates reported in other samples. For example, in another sample of MS caregivers, Akkus [18] reported a mean ZBI score of 36.42 (SD = 18.41), while Buchanan and Huang [64] found that 40% of their sample described caregiving as burdensome some of the time and 11.4% reported that caregiving was burdensome all of the time. These findings suggest the need for health care providers to continuously assess the mental health of caregivers and to provide them with access to services that can include emotional support (e.g., support groups, volunteer organizations, nursing care, spiritual/religious leaders, and communities) throughout the disease's duration.

### 4.1. Limitations and Future Research

The findings of this study should be viewed in light of several limitations, which can be considered potential areas for future research. Unlike many samples of MS caregivers, caregivers in the current study were predominately women who were mothers of the care recipients. This is indeed a rarity in the MS literature, as generally caregivers are male spouses providing care for their wives with MS. Future studies should examine the influence of gender roles prominent in Latino cultures on caregiving behaviors in both women and men MS caregivers and how these roles might play out in same-gender and opposite-gender caregiving relationships.

Because of the strong cultural values of familism and the stigma associated with neurological illness, caregivers within this sample may have underreported symptoms of burden, depression, and anxiety. Given the desire to fulfill cultural roles, Latina women, especially mothers, may not perceive caregiving as burdensome or they may be reluctant to disclose feelings of strain, anxiety, and sadness. By contrast, in other countries where male spouses or romantic partners typically fulfill MS caregiving roles, perceptions of burden may be stronger, as caregiving is a new skill set for them.

Additionally, the reported household incomes of the families in this sample may be higher than other caregivers who do not receive subsidized care and who are not employed while providing care. This study's participants were recruited from an urban university medical center and a local chapter of the MS foundation. As such, this sample's utilization of care and access to resources may be greater than most caregivers, especially those living in rural areas or individuals living in underdeveloped societies where health care is not as accessible. The findings in this sample may overlook or underestimate the true needs and psychosocial functioning of caregivers who do not have adequate resources and thus cannot acquire medical care for their loved ones and are not as well connected with community-based organizations.

Unmet family needs was one of the most important constructs assessed in the current study. At present, there are few empirically validated neurological-specific family needs assessments for use in Latin America other than that used in the current study. As such, the FNAT was used because the measure had been validated in a sample of caregivers of individuals with neurological conditions in Latin America [50]. Despite this strength, this measure's limitations include a narrow assessment of additional possible family needs which may or may not be MS-specific. As administered in the current study, only one individual in the family completed the questionnaire. However, in order to truly assess family needs, administration of the measure should include reports from all family members of the individual receiving care. As the measure is currently written and administered, the results only offer the perspective of one reporter but attribute this variance to the perspectives of others within the family unit. By including multiple reporters, researchers can then differentiate between variance observed within reporters (unique reporter variance) and across reporters (e.g., shared variance).

Another way to improve the measure is to increase the comprehensiveness and specificity of the constructs being assessed. Although the items in the current measure come from an aggregate of items from needs assessments of caregivers of individuals with MS and other neurological disorders, the current measure does not include items that assess potentially important aspects such as the need for respite care, the specific types of information that caregivers need, assistance with obtaining medical equipment, and the need for holistic or nontraditional medical practices that may be common and useful in Latin America. Including these items may come from additional focus groups and surveys with MS caregivers in other communities, especially communities of individuals who are hard to reach or who do not regularly access medical care. As such, the literature could greatly benefit from an MS-specific measure of family needs that has been developed using a Spanish-speaking sample and has been empirically validated in similar samples.

Similarly, the current study used the MS Impairments Questionnaire, a 30-item checklist of MS symptoms developed by Knight et al. [26] to assess the types of symptoms that patients experience. Although this questionnaire assesses the general clusters of symptoms shown to be common in individuals with MS, the measure itself has not gone through extensive psychometric evaluation. Additionally, several of the items comprising this questionnaire are vague and may not translate well into Spanish (e.g., “being unreliable”). Because of the importance of accurate assessment of MS symptoms, this measure should undergo further revision and evaluation in an attempt to assess additional symptoms of MS, as some of the common symptoms such as sexual dysfunction, attention problems, and heat sensitivity were omitted. A revised version should also include a factor analysis of items, as well as measure of disease severity or disability since the presence of a symptom does not necessarily indicate its functional impact or severity. As with the FNAT, a measure of MS symptoms should include multiple raters and/or a review of medical records to support self-reported and caregiver-reported data. Relying on the caregiver's perspective may overlook symptoms that the caregiver is unaware of (e.g., sexual dysfunction) and result in an incomplete assessment of patient functioning.

Finally, although the assumptions of normality were met for the SEM in the current study, the sample size was too small to generate accurate fit indices. Though they were presented for reference, they likely lack the stability that would occur with sample sizes greater than 200. As a result, these fit indices should be interpreted with extreme caution, and similar models should be run with larger samples in order to more accurately assess whether the current model or other models better fit the patterns in the data.

## 5. Conclusions

This study provided empirical support for the mediational role of unmet family needs in the relationship between MS impairments and caregiver mental health in Guadalajara, Mexico. These findings suggest that MS impairments may affect both the individual caregiver and the family unit. As a result, MS rehabilitation interventions, especially in Mexico and other Latin American countries, should comprehensively assess and target the patient's functioning, the family's unmet needs, and the caregiver's mental health functioning. Doing so—if supported by future research—could improve services for a population that has faced marginalization and a dearth of care within traditional rehabilitation settings.

## Figures and Tables

**Table 1:** Characteristics of MS caregivers (*n* = 81).

Demographic variable	Value
Age, years, mean (SD)	43.37 (15.32)
Sex, %	
Female (*n* = 54)	66.7%
Male (*n* = 26)	33.3%
Years of education, mean (SD)	11.74 (4.42)
Marital status, %	
Married or partnered (*n* = 55)	67.9%
Single (*n* = 19)	23.5%
Widowed (*n* = 4)	4.9%
Divorced or separated (*n* = 3)	3.7%
Relationship to individual with MS, %	
Parent (*n* = 37)	45.7%
Spouse/romantic partner (*n* = 26)	32.1%
Sibling (*n* = 10)	12.3%
Child (*n* = 5)	6.2%
Friend (*n* = 1)	1.2%
Professional caregiver (*n* = 1)	1.2%
Other (*n* = 1)	1.2%
Duration of caregiving	
Number of months, mean (SD)	52.31 (59.29)
Hours per week of care, mean (SD)	70.96 (60.66)
Current occupation, %	
Homemaker (*n* = 25)	30.9%
Full-time employment (*n* = 21)	25.9%
Part-time employment (*n* = 19)	23.5%
Student (*n* = 7)	8.6%
Unemployed (*n* = 4)	4.9%
Retired (*n* = 3)	3.7%
Other (*n* = 2)	2.5%
Monthly household income, %	
Less than minimum wage (*n* = 1)	1.2%
Minimum wage (*n* = 6)	7.4%
1-2 times minimum wage (*n* = 11)	13.6%
2-3 times minimum wage (*n* = 10)	12.3%
3-4 times minimum wage (*n* = 7)	8.6%
4-5 times minimum wage (*n* = 11)	13.6%
More than 5 times minimum wage (*n* = 35)	43.2%

**Table 2:** Characteristics of individuals with MS as reported by caregivers (*n* = 81).

Demographic variable	Value
Age, years, mean (SD)	33.25 (10.78)
Sex, %	
Female (*n* = 56)	69.1%
Male (*n* = 25)	30.9%
Years of education, mean (SD)	13.34 (3.97)
Marital status, %	
Single (*n* = 40)	49.4%
Married or partnered (*n* = 36)	44.4%
Divorced or separated (*n* = 5)	6.2%
MS clinical course, %	
Relapse remitting (*n* = 64)	79.0%
Secondary progressive (*n* = 16)	19.8%
Primary progressive (*n* = 1)	1.2%
Age of symptom onset, mean (SD)	26.29 (9.76)
Age at diagnosis, mean (SD)	28.17 (10.17)
Current occupation, %	
Full-time employment (*n* = 22)	27.2%
Homemaker (*n* = 19)	23.5%
Part-time employment (*n* = 15)	18.5%
Student (*n* = 11)	13.6%
Unemployed (*n* = 6)	7.4%
Receiving disability (*n* = 7)	8.6%
Other (*n* = 1)	1.2%

**Table 3:** Summary of MS impairments reported by caregivers (*n* = 81).

Impairment domain	Impairments endorsed	% endorsing impairment	Number of patients with observed impairments
Neurological	Tiring easily	79%	64
	Paralysis	69%	56
	Poor eyesight	62%	50
	Loss of sensation	54%	44
	Clumsiness	52%	42
	Pain	36%	29
	Incontinence	27%	22
	Seizures	14%	11

Emotional	Depression	68%	55
	Easily upset	68%	55
	Irritability	58%	47
	Mood changes	58%	47
	Anxiety	49%	40
	Loss of interest	33%	27

Functional	Difficulty walking	69%	56
	Doing things slowly	56%	45
	Trouble reading	33%	27
	Difficulty writing	32%	26
	Difficulty talking	27%	22
	Difficulty eating	22%	18
	Difficulty hearing	20%	16

Cognitive	Forgetfulness	62%	50
	Difficulty concentrating	53%	43
	Difficulty thinking	38%	31
	Poor decision making	30%	24
	Difficulty learning	27%	22
	Denying problems	27%	22

Behavioral	Acting impulsively	35%	28
	Upsetting other people	28%	23
	Not being reliable	12%	10

**Table 4:** Summary of unmet family needs (*n* = 81).

Family need	% endorsed as unmet	Number of caregivers reporting need	Domain
I need complete information.	71.6%	58	Information
I need specialized information about the patient.	70.3%	57	Information
I get help from the community (reverse coded).	65.5%	53	Social support
I get support from my church (reverse coded).	61.7%	50	Social support
I need to discuss my feelings with someone who has been through the same experience.	45.7%	37	Information

**Table 5:** Summary of caregiver mental health variables.

Variable	Value
PHQ-9 total score, mean (SD)	5.92 (5.27)
Mild depression (%)	26%
Moderate depression (%)	16%
Moderate–severe depression (%)	3.7%
Severe depression (%)	1.2%
STAI total score, mean (SD)	47.01 (21.40)
STAI state, mean (SD)	22.67 (11.82)
STAI trait, mean (SD)	24.34 (10.97)
State moderate anxiety (%)	32%
State severe anxiety (%)	2.5%
Trait moderate anxiety (%)	31%
Trait severe anxiety (%)	3.7%
ZBI total score, mean (SD)	22.02 (14.72)
Mild to moderate burden (%)	29.6%
Moderate to severe burden (%)	12.3%
Severe burden (%)	1.2%
SWLS total score, mean (SD)	23.43 (6.35)
Life dissatisfaction (%)	26%
Neutral (%)	7%
Life satisfaction (%)	67%
